# Investigation of a Combined Surveying and Scanning Device: The Trimble SX10 Scanning Total Station

**DOI:** 10.3390/s17040730

**Published:** 2017-03-31

**Authors:** Elise Lachat, Tania Landes, Pierre Grussenmeyer

**Affiliations:** ICube Laboratory, Photogrammetry and Geomatics Group, National Institute of Applied Sciences (INSA), 24 Boulevard de la Victoire, 67084 Strasbourg CEDEX, France; tania.landes@insa-strasbourg.fr (T.L.); pierre.grussenmeyer@insa-strasbourg.fr (P.G.)

**Keywords:** Terrestrial Laser Scanning, robotic image-assisted total station, trimble SX10 scanning total station, surveying and scanning projects, geometric investigations, comparisons

## Abstract

Surveying fields from geosciences to infrastructure monitoring make use of a wide range of instruments for accurate 3D geometry acquisition. In many cases, the Terrestrial Laser Scanner (TLS) tends to become an optimal alternative to total station measurements thanks to the high point acquisition rate it offers, but also to ever deeper data processing software functionalities. Nevertheless, traditional surveying techniques are valuable in some kinds of projects. Nowadays, a few modern total stations combine their conventional capabilities with those of a laser scanner in a unique device. The recent Trimble SX10 scanning total station is a survey instrument merging high-speed 3D scanning and the capabilities of an image-assisted total station. In this paper this new instrument is introduced and first compared to state-of-the-art image-assisted total stations. The paper also addresses the topic of various laser scanning projects and the delivered point clouds are compared with those of other TLS. Directly and indirectly georeferenced projects have been carried out and are investigated in this paper, and a polygonal traverse is performed through a building. Comparisons with the results delivered by well-established survey instruments show the reliability of the Trimble SX10 for geodetic work as well as for scanning projects.

## 1. Introduction

As with almost every research area, the field of surveying and its equipment evolve with the technological innovations leading to ever faster, more accurate and more versatile devices. Not only land surveyors but also specialists of various research communities make use of surveying techniques. These can be applied for tasks varying from detection of changes to structural monitoring, whether in geosciences or for industrial applications. Depending on the scale of the surveyed area and on the kind of data required for the analysis, various surveying technologies can be employed. Next to large-scale remote sensing methods that are more suited for covering wide study areas, geodetic measurements through Global Navigation Satellite Systems (GNSS), total stations or Terrestrial Laser Scanners (TLS) are commonly applied. Whereas individual points of interest are selected by the user who guides their direct measurements while using GNSS or total stations, TLS provide dense point clouds measured along a predefined frame and representing the 3D geometry of a scene surface. The 3D coordinates of points of interest are then indirectly obtained through point cloud processing. Due to the development of ever more powerful stereo-matching algorithms, photogrammetry methods also lead to the creation of dense point clouds.

As regards total stations, over the last fifty years this specific equipment has known drastic changes. From manual tacheometers to motorized robotic total stations, the electronics have brought numerous new facilities to surveyor instrumentation. Nowadays, almost every total station is capable both of performing fast reflectorless measurements and of prism autotracking. Besides, steering the total station through one or more cameras integrated into the hardware is possible and prototypes were studied in the 1990s as in [1]. The adjunction of a GNSS receiver is also possible on some current instruments. In fact, total stations now tend to become multi-task devices composed of several built-in sensors. A comprehensive review of the total station evolution is proposed in [2].

Parallel to these developments, in recent decades TLS have gained wide acceptance and their use is more and more generalized for surveying and monitoring tasks. In some cases, TLS even tend to become optimal alternatives to total stations [3,4], even if the latter remain valued devices. Thanks to ever more accurate and faster sensor technology, as well as always deeper data processing software capabilities, terrestrial laser scanning has now been proved as a reliable technique for high accuracy, 3D mapping and documentation of physical scenes and structures [5,6]. Besides, the research topics around scanning technology are still growing, regarding for example the geometric accuracy of the produced data [7] or the influence of different parameters [8]. However using most of the TLS, the dense point clouds captured are known in a local reference system related to the scanner. Thus conventional surveying techniques are still required to achieve what is known as the georeferencing of the project. To take advantage of both geodetic measurement principles as well as scanning facilities, the trend is now to combine a laser scanning module within the total station hardware to build a unique device. The recent Trimble SX10 scanning total station [9] released in October 2016 belongs to this category of innovative and versatile multi-sensor systems.

This article aims at observing and assessing the Trimble SX10 capabilities by means of experiments carried out based on its different measurement modules. To put the recent device in the context of modern total stations development, it is first compared to recent total stations. The proposed review is illustrated by examples of case studies applying these techniques. A second part is dedicated to the investigation respectively of the Electronic Distance Measurement (EDM) unit (Section 4), and of the scanning module (Section 5). The idea is to focus on real projects which have been formerly handled using other instruments, but for which the use of the Trimble SX10 would represent an added value during the survey. The collected datasets have thus been compared to the results obtained with conventional total station and Terrestrial Laser Scanner.

## 2. Modern Total Stations—State of the Art and Case Studies

Before using the Trimble SX10 in various projects to investigate the geometric quality of the data it delivers, in this section some of its technical specifications are confronted to those of other instruments offering similar functionalities.

### 2.1. Overview of Image-Assisted and Scanning Total Stations

Despite the diversity of surveying instruments available to land surveyors, the total station is still a staple instrument in recording punctual and precise measurements. From the initial theodolites allowing the user to measure horizontal and vertical angles, to the instrument known as tacheometer or total station including an Electronic Distance Measurement (EDM) unit to record distances, the electronics have contributed to the improvement of hardware. Today total stations are moving towards robotic multi-sensor systems which combine ever more sophisticated functionalities. Thus the broad range of instruments available on the market may appear staggering, as described in the recent report in [10]. Scherer and Lerma [2] provide a comprehensive and thorough review about the historic and constant evolution of this technology, as also do [11]. It shows that not only the EDM has evolved, allowing for example reflectorless distance measurements, but also the adjunction of built-in sensors has contributed to changing the hardware. In fact, most of the current total stations are robotic and motorized, achieving among other features the automatic recognition and tracking of reflector prisms or the remote steering of the device by the user. All these new functionalities offered by total stations have triggered further improvements and innovations in these surveying instruments.

Total stations which are equipped with one or more built-in digital camera(s) for targeting and steering operations are reported in the literature as Image-Assisted Total Stations (IATS), see for example [2,12,13]. Even if the idea of combining theodolite and camera does not have a recent origin as explained in [2], the first prototypes of a modern device combination were constructed in the 2000s. Prototypes applying this major improvement are described notably in projects from universities [1,12,14,15,16]. While dealing with such an instrument, its calibration is highly important. Calibration models for the on-board cameras are described in [12,17,18]. The calibration ensures the perfect coaxiality between the built-in camera and the telescope line of sight, so that the observed image corresponds to the targeted area containing the point to be measured. Ehrhart and Lienhart [18] specifically analyze different error sources related to the use of an IATS as well as their impact on experimental geodetic network measurements.

More recently a few manufacturers of surveying instruments have introduced scanning functions into their total station hardware. This innovation was driven by the generalized use of TLS in surveying activities, and made possible by the combination of instrument motorization and reflectorless distance measurement. First releases of such modern total stations offer some pre-built scanning modules such as line or grid scanning. This is the case of the Topcon Imaging Station series launched on the market in 2008 and illustrated by the Topcon IS-3 [19] in Table 1. In addition to imaging facilities and the possible creation of 360° panoramas, this instrument also offers simple scanning functions with a measuring rate lower than 20 points per second. This means that points are captured in a defined window area along a grid interval specified by the user, but without user intervention during measurements and in particular no need to aim at each individual point.

There is quite a significant gap between scanning rates of total stations such as the previous Trimble VX Spatial Station or more recently Trimble S9 (Trimble Inc., Sunnyvale, CA, USA) (15 pts/s) but also Topcon IS-3 (Topcon Positioning Systems Inc., Livermore, CA, USA) (20 pts/s, see Table 1), and scanning rates of current TLS which are able to capture millions of points a second. This gap was slightly reduced as Leica introduced its Nova series total stations. The Leica Nova MS50 (Leica Geosystems, Heerbrugg, Switzerland) was launched in 2013 and a new release called Leica MS60 has been marketed since 2015 [20]. They present the same scanning speed of about 1000 points per second as indicated in Table 1. The very latest release of Trimble SX10 in October 2016 shows a promising improvement since it offers an increased scanning rate of 26,600 points per second [9]. Further specifications of Topcon IS-3 (Topcon Positioning Systems Inc., Livermore, CA, USA), Leica MS60 (Leica Geosystems, Heerbrugg, Switzerland) and Trimble SX10 (Trimble Inc., Sunnyvale, CA, USA) are listed in Table 1. Besides, because the capability of total stations to feature scanning facilities is a quite recent improvement, the names given by manufacturers or stated in the literature for this technology can vary. Leica often calls its instruments of Nova series *multi-stations*, whereas Trimble refers to SX10 device as a *scanning total station*. Since the second acronym is more general, it will be used in the remaining parts of this article to refer to this technology.

Wagner [13] presents the specifications of two other modern total stations in a table similar to Table 1. When having a look at these two tables, it appears that all major surveying instrument manufacturers (Leica, Trimble and Topcon among others) currently develop IATS which integrate some scanning functions. Nevertheless regarding scanning specifications and more particularly the scanning rate, one can say that actually only Leica MS50 or MS60 and Trimble SX10 total stations are capable of efficient scanning. A big advantage of these devices is the possibility to measure all point coordinates in a same unique reference system. It should be noticed that, nowadays, the high difference of scanning rate between scanning total stations and TLS is mainly due to the mass of the total station telescope that has to be moved during the scanning process [2,13]. The considerably higher measuring speed of Trimble SX10 compared to Leica MS60 is due to the absence of telescope on the SX10 device, which is one of the most original features of this instrument. Since most of the total station users are used to manually aiming by looking through a telescope, this is a daring decision of the manufacturer which clearly benefits the scanning rate. As explained later in Section 3.1, the telescope functionality is replaced by the presence of cameras and the use of a remote controller shown in the corresponding picture in Table 1.

### 2.2. Related Work

A literature review shows that IATS and scanning total stations have been adopted in several fields which usually apply conventional tacheometry. The advantages related to the use of modern devices is often underlined in these papers. Unfortunately most of the time contributions do not focus on the assessment of the devices but rather on their use in specific case studies, except when considering contributions related to prototypes of IATS. This is even more true for scanning total stations which were released later on. Besides, it appears that scanning total stations are sometimes only applied for the cameras they contain. In the following subsection an overview of contributions from different application fields using IATS and scanning total stations is proposed. It mainly encompasses contributions where the devices of Table 1 were used.

A first application field where the results are promising deals with the monitoring of civil engineering structures. Examples of bridge monitoring [21], dam monitoring [22] as well as chimney deformation monitoring [23] are reported. The main advantage mentioned of IATS is the possibility of automatically detecting targets thanks to the camera-based views without the necessary use of reflective prisms, as do [21] to investigate vibrations and displacements on a footbridge. While this previous work only uses the camera capabilities of the Leica MS50 to prevent structural failure, in [22] the grid scanning module of this same device was used to model a dam surface. The dam surface was scanned as well as specific cross-sections using the line scanning module to carry out subsidence analysis during loading experiments on the dam. It appears that the data captured by the scanning total station was valuable to investigate the overall deformation behavior since the acquired point clouds enabled the identification of different deformation patterns. For industrial chimney deformation monitoring, Zheng et al. [23] use a Topcon IS to capture oriented images from several stations distributed in a network around the chimney. Outlines of the chimney are extracted from these pictures after different image processing steps, and a model of the surveyed object is finally reconstructed. A last example related to civil engineering reports on the survey of reservoirs with UAV photogrammetry [24]. To validate the photogrammetry-based model, a reference model of the reservoirs is created thanks to a Leica MS60. This kind of instrument appears as a suitable solution to the authors since a station network is necessary to obtain a complete model thanks to multiple scan stations.

In the field of geosciences, deformations or changes of natural areas are often monitored using GNSS and conventional tacheometry. An example of a project whose aim is the development of an early warning system for alpine instable slopes is presented in Thuro et al. [25]. In this context, monitoring of landslides is performed thanks to an IATS prototype based on a conventional Leica total station. The authors use this prototype since the control of the mounted camera with external devices is required. Indeed the video frames are used for the automatic detection of natural targets such as surface rocks or debris in this case, rather than artificial marks or reflectors used in conventional methods. A flowchart that recaps the steps of natural or artificial target detection with an IATS is also proposed in [25]. Looking at a similar topic, Gomez-Vasconcelos et al. [26] do not only use pictures captured by a Leica Nova MS50 but also the georeferenced point clouds it delivers to investigate tectonic areas. The authors explain that after meshing the point clouds they are able to create a digital outcrop model, and fault displacements are measured within this model. This data is combined with information coming from other sensors in order to predict potential interactions between volcanism and seismic hazards in these areas. Then regarding change detection, erosion of a beach is surveyed in [27]. The changed areas within a determined time interval are studied thanks to the grid scanning module of a Topcon IS. Wagner [13] describes an application example of geo-monitoring that is also related to the study of changes. In this application, displacement vectors between two periods in a mountainous terrain have been computed thanks to a Leica MS50. In such a challenging terrain, the authors highlight the advantage of carrying only one surveying instrument up to the observation point.

Some contributions report the use of modern total stations for building survey and cultural heritage applications. Wozniak et al. [28] present results obtained for the creation of a building facade 3D model using data from a Topcon IS-3 as well as images acquired with a conventional digital camera. In this study aiming at documenting geometrical features of buildings, the Topcon Imaging Station is used in its standard scanning mode for reflectorless point acquisition. Despite a stated advantage of requiring one unique device capable of multiple survey tasks, some main limitations of the device are underlined, such as the low measurement speed or the low image resolution. In the context of Building Information Modeling (BIM), Sepasgozar et al. [29] relate on mobile and static acquisition methods for as-built modeling, given that point clouds are increasingly used as data in construction. Static acquisitions performed with a Leica MS50 are compared with data obtained based on mobile technologies, providing a better spatial accuracy than mobile data, although requiring more time. The same Leica MS50 is used to provide reference data in a similar approach described in [30]. In this second work the authors aim at evaluating a mobile mapping system on a larger scale test field, in which control points on building facades and street profiles were previously captured with the multi-station. Some more examples rather related to cultural heritage are reported in [31,32] which both use the Topcon IS. Evgenikou and Georgopoulos [31] investigate the 3D modeling of small artifacts thanks to different sensors, and a brief overview of results obtained for architectural survey of a bridge are shown in [32]. Even if point density seems to be the major issue in both cases, the device could be adapted for documenting objects of similar size as a bridge whereas it is visibly not adapted for small artifacts reconstruction.

Metrology applications can finally be reported, considering leveling capabilities with a Leica MS60 [33], or collimation measurements with an experimental camera module on a total station [14].

## 3. Trimble SX10 Technical Properties

Because of the recent release of the Trimble SX10 scanning total station, no scientific review relates to its use yet. The purpose of this section is to describe and to verify some of the announced device specificities by focusing mainly on its cameras and scanning modes.

### 3.1. Overview of Built-In Digital Cameras

Among the specificities of the SX10 device, the camera technology is particularly developed since three different calibrated cameras are built into the rotating part of the hardware. Unlike in previous IATS from Trimble (e.g., Trimble S9), not only a wide-angle camera is available but also two other cameras with smaller fields of view. According to manufacturer documentation, these cameras are called *overview*, *primary* and *telescope* cameras. The overview or wide-angle camera as well as the primary camera are mounted parallel with the EDM axis but with an offset. The telescope camera located in the path of measurement axis is introduced for magnification. Each camera chip has a 5 megapixel resolution (i.e., 2592 × 1944 pixels), but given their different fields of view the pixel size differs for each camera as described in Table 2.

Since there is no true telescope in the hardware, optical sighting is no longer foreseen and is replaced by the use of digital cameras. The device can thus only be controlled remotely through the video stream displayed on an external tablet or a remote controller, given that the hardware does not contain any screen as visible in the figure of Table 1 in the previous section. The field software Trimble Access is used for controlling the device in each kind of operation. After coarsely aiming the device at a survey point, navigation within the video is ensured thanks to the overview and primary cameras which both offer two zoom levels. The user just has to tap on the touch-sensitive screen to more finely aim at the survey point by overlaying digital cross-hairs with the target. Zoom in for final accurate targeting is then enabled by the telescope camera. The different zoom levels offered through the three cameras are also useful while framing an area to be scanned.

Not only steering of the device and point measurement are possible with the innovative on-board camera technology used. In many cases, it can be interesting to document the project by taking pictures of the measured points or targets as a reminder. Some notes and comments can then be added on the picture through the touchscreen. When opening an exported project in one of the manufacturer provided office software, the pictures captured during field operations are automatically referenced to the station from which they were taken, as well as to the measured point they correspond to. Pictures can be exported for further office applications such as for example post-acquisition documenting. Because of the pixel size offered by the primary camera, it would be interesting in the future to investigate its use in photogrammetry projects. However, the limited resolution of the acquired pictures might be restrictive for such approaches.

**Figure 1:**
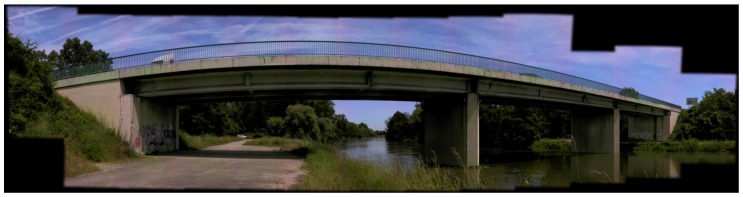
Example of a panoramic image captured with Trimble SX10 on a bridge using the primary camera and an overlap of 20% between individual pictures.

Another interesting feature is the creation of panoramic images. By selecting an area on the screen, imaging total stations can capture several overlapping pictures in the horizontal and vertical directions. The field software Trimble Access offers the possibility to choose the camera that should be used to create the panorama, as well as the overlapping rate between pictures. When the pictures are captured within a geodetic network of known points, the imaging data is directly geolocalized. An example of a panorama performed on a bridge is shown in Figure 1. In addition, the user can choose between the overview and primary camera for point cloud colorization in scanning projects, depending on the appropriate acquisition time and texture quality for the project considered.

It should be noted that the station setup uses a fourth camera for centering the device above the reference point. A plummet camera with a fixed focus and a 6° field of view replaces the optical or laser plummet. This camera-based solution may be confusing for uninitiated users at the beginning. Nevertheless this solution offers a ground pixel size of about 0.2 mm at a 1.55 m instrument height with a quoted accuracy of 0.5 mm at this same height according to the manufacturer.

### 3.2. Specifications of SX10 Scanning Modes

The Trimble SX10 has been devised as an innovative instrument combining the capabilities of a total station with those of a powerful laser scanner. Due to its particular design and to its capacities, it can even be seen as a TLS with total station measurement facilities rather than the contrary. In fact, the Trimble SX10 scanning rate tends to approach the scanning rate of a conventional TLS as exposed in Table 1. This subsection seeks to describe the scanning module contained in the device.

The Trimble SX10 makes it possible to acquire dense point clouds of a scene in a very timely manner. The area to be scanned can be directly selected by the user without the necessity to perform a 360° preview point cloud for the selection beforehand. This area is defined through tapping on the touchscreen between four possible specificities. Full dome acquisition of dense point clouds can be performed to obtain a global overview of the surrounding environment with a maximal field of view of 360° × 300°. But the user can also choose to select a viewing window according to three possible kinds of frames: a band defined by two points placed on the upper and lower limits, a rectangle defined by two points or a polygon for more complex shapes. After framing the area of interest to be scanned, other settings can be modified as for example the point density. The four point densities correspond to 1, 2, 4 or 8 slightly shifted passes of the scanning line from coarse to superfine density, and the corresponding point spacings are listed in Table 3. A last setting to parametrize is the choice of the camera that is used to colorize the point cloud, as well as the overlap between these pictures.

A valuable asset while using the SX10 for point cloud acquisition is the possibility of visualizing the acquired data in the field. With the Trimble Access software a review of the captured point clouds is possible and they can be displayed either in true color, with intensity values or with one color per scan according to the scan station it is related to. Zoom in on the captured point cloud through the tablet is also foreseen, which is valuable in the field to ensure there are no missing parts in the surveyed area.

The scanning function presented in this subsection is assessed later through a set of comparisons made between some SX10 produced point clouds and datasets captured with other TLS. In this context, Table 4 gives an overview of some major specificities of Trimble SX10 as well as those from laser scanners used later in this article. It appears that the Trimble SX10 scanning module offers a wider measurement range compared to FARO Focus^3D^ (FARO Technologies, Lake Mary, FL, USA) [34] and Leica ScanStation C10 (Leica Geosystems, Heerbrugg, Switzerland) [35]. The FARO Focus^3D^ is based on phase-shift measurement principle unlike the two other time-of-flight laser scanners, which explains the high scanning rate difference. More recent ScanStations from Leica exist with increased performances in terms of precision and scanning rate, however the C10 was the most recent laser scanner from Leica available in our equipment.

### 3.3. Practical Investigation of Maximal Measurement Range

**Figure 2:**
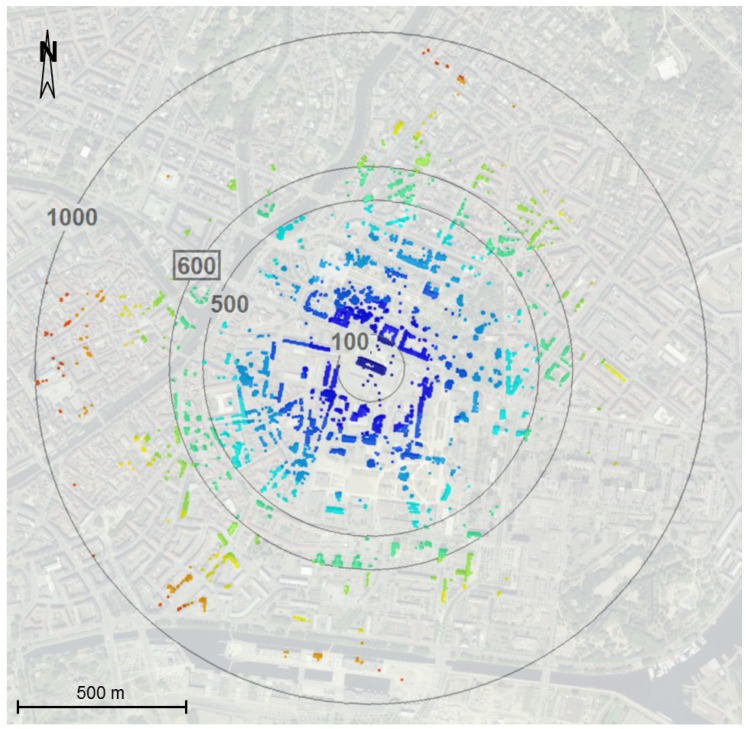
Top view of a 360° point cloud acquired in band scanning mode from a building roof. The point cloud is colorized according to range and significant ranges are represented.

In order to assess the maximal measurement range announced by the manufacturer, an outdoor experiment was carried out. To ensure a large visibility, the tests were performed from a building roof surrounded by other buildings in the city center of Strasbourg, France. A 360° band-shaped point cloud was acquired from the roof with a low point density to keep a moderate scanning duration. Figure 2 shows a top view of the acquired point cloud. Centered on the scanner location which is known, circles have been drawn with rays representing different acquisition ranges. The color codification represents the points divided per 100 m range. In Table 5, the number of points belonging to each 100 m portion is reported, as well as the percentage it represents with respect to the number of points of the whole point cloud.

It appears that the furthest point detected is about 900 m from the scanner location, which means that the announced maximal measurement range of 600 m is well respected. However, the farther the points are located, the lower the point density. Indeed, points acquired beyond the announced maximal range are more scattered. This is confirmed while cumulating the proportion of points contained within the first intervals and listed in Table 5. About 99% of the total amount of points are concentrated before 600 m range. The last portions of points between 600 m and 900 m count about 14,000 points, which represents only 1% of the total amount of acquired points. Besides, the geometry of the buildings located at this range is hardly distinguishable, since most of the time only a few points were acquired on these faraway buildings.

## 4. Investigation of the EDM Unit and General Surveying Functionalities

The Trimble SX10 scanning total station can be seen as a hybrid solution. Since the device offers more than the powerful laser scanning functionalities described in Section 3.2, it also belongs to the category of total stations. One can even speak of an Image-Assisted Total Station because of the cameras it contains as described in Section 3.1. In this section the use of total station functionalities is introduced and illustrated by a network survey carried out in a building.

### 4.1. Total Station Working Principle

Standard surveying solutions are included in the SX10 device. Regarding station setup, similar options to those present in conventional tacheometers are available. Depending on the existing references on the field, the user can choose between resection or stationing on a known point with orientation on one or several reference(s). Measurements can be automatically repeated in both telescope positions through direct and reverse angle measurements. The user can define beforehand which points should be observed twice, such as references or control points for example. Besides, numerous coordinate geometry (known as COGO) computations are handled by the device. These functionalities include point coordinates calculations, but also intersection computations or point and line stakeout among other features. Station setup as well as COGO computations are controlled through the field software Trimble Access during surveying and scanning projects.

All the measured data are integrated and stored into a same job created in the Trimble Access software. A very useful functionality offered by the software interface is the real-time viewing of the acquired data directly on the tablet or controller screen. The whole project and thus topographic, optical and scanning data it contains can be displayed all together. This helps in the field by making it possible to control the potential missing areas that still have to be captured.

Another particularity while using the Trimble SX10 as a total station is the presence of a camera system. As a matter of fact, manual sighting is no longer foreseen because of the absence of an optical telescope on the device. The user thus steers the instrument through real-time video streaming. Movements of the sighting axis to aim the instrument towards the targeted point are directed by tapping directly on the screen. As during optical sighting, coarse and fine targeting are necessary to accurately reach out to the target. Coarse targeting is first achieved using the overview and primary cameras. Accurate targeting is performed in a second step thanks to the telescope camera which offers a better image magnification. This process is detailed in Section 3.1 related to on-board cameras.

### 4.2. Geodetic Traverse Survey

**Figure 3:**
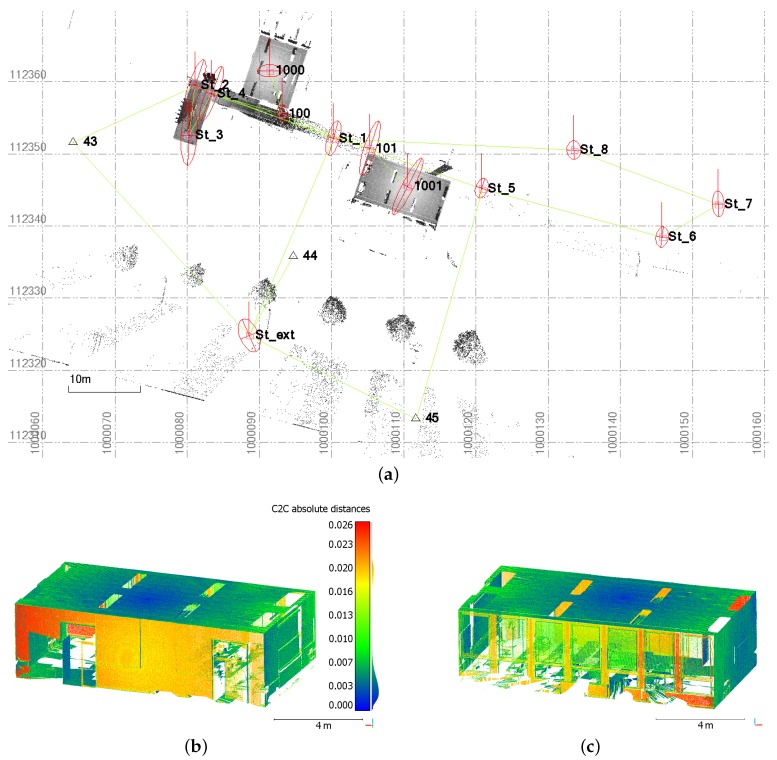
(**a**) Top view of the network performed within a building with error ellipsoids computed after adjustment for each network point. The point clouds acquired during this project appear in grayscale. (**b**) and (**c**): Visualization under two different views of cloud-to-cloud distances computed between point clouds of the room acquired from station 1001, before and after network adjustment. Deviations unit: meter.

The surveying solutions proposed by Trimble SX10 device have been investigated through the achievement of a geodetic closed traverse. The measurements were performed through a building and some point clouds have been acquired on rooms along the traverse. Topographic data are displayed together with captured point clouds in Figure 3a. This traverse begins outdoors at the building ground floor level on an unknown point, using references to determine its coordinates through resection. It then passes through the first floor before closing on the first survey point located on the ground floor. This is the reason why the measurements appear to be superimposed on the top view as proposed in Figure 3a. A prism fixed on a tripod was used to signal each network point. Laser scanning survey as well as traverse measurement were carried out within the same day, and the final loop closure of the traverse is useful to control the coordinates of the two last measured points in this particular case. The fact that the traverse is closed also enables the computation of the adjusted coordinates of the measured points.

Both field and office applications have been experimented during this project, using the manufacturer office software Trimble Business Center (TBC) for data post processing. This software offers several facilities including visualization of the acquired data but also adjustment computations. Figure 3a is a screenshot obtained from TBC and shows the considered network points which are linked. Since measurements on references have been carried out from some traverse points, the network adjustment tool was chosen to compute the adjusted values of the point coordinates. In this particular project, the standard deviations do not exceed 8 mm for all adjusted network points with a mean value of about 4 mm. Error ellipsoids are also computed for each network point during adjustment computation in order to provide an idea of point precision after adjustment. They can be plotted together with the points they correspond to, as can be seen in Figure 3a. This figure illustrates that error ellipses in the planar view are larger particularly for points obtained through polar measurements (points 101, 1000 and 1001). The knowledge of these statistic values provides valuable information for further inspection and confidence analysis of the project.

Network adjustment has been recomputed using another software dedicated to the adjustment of geodetic networks (Covadis, Geomedia). Since the deviations between the final coordinates adjusted by this means and through TBC never exceed 1 cm, the results obtained thanks to the manufacturer software can be validated. Besides, network adjustment has a direct influence on the coordinates of point clouds contained within the project. To illustrate this influence, the whole point cloud acquired during the project has been exported twice, first as raw data and then after network adjustment. The distances computed between both point clouds focusing on one room are shown from two opposite points of view in Figure 3b,c. The cloud-to-cloud comparison has been performed using the dedicated free software CloudCompare [36]. Deviations no larger than 2.5 cm and with a mean value of about 5 mm are observed. It also appears that the larger deviations are predominantly visible on two opposite faces of the room, which means that the offset between both datasets mainly occurs along one direction. Their order of magnitude near to 1 cm almost corresponds to the deviation between station coordinates before and after adjustment. These deviations caused by the polar measurement of successive points within a traverse attest to the adjustment influence on scanning data.

## 5. Assessment of Laser Scanning Projects

Some laser scanning projects have been conducted with the Trimble SX10, whereby different objects and georeferencing methods were considered. When the coordinates of reference points are known in the project area, a direct georeferencing of the acquired point clouds can be performed. This consists in the capture of point clouds which are directly known in a required reference system. Due to surveying facilities included in the Trimble SX10 device, the scanning projects it provides may be directly georeferenced without supplementary material. On the other hand, georeferencing can be performed as a post-processing step, consisting of the transformation of all points of the data into the required geodetic coordinate system. This process, known as indirect georeferencing is often made based on the measurements of targets with known coordinates in the destination system [37].

Two field cases are presented to illustrate this section. The first one is a church facade scanned from three different setup locations without consideration of georeferencing, whereas the second field case is a bridge scanned from six different setup locations and directly georeferenced.

### 5.1. Investigation of a Building Facade Survey

**Figure 4:**
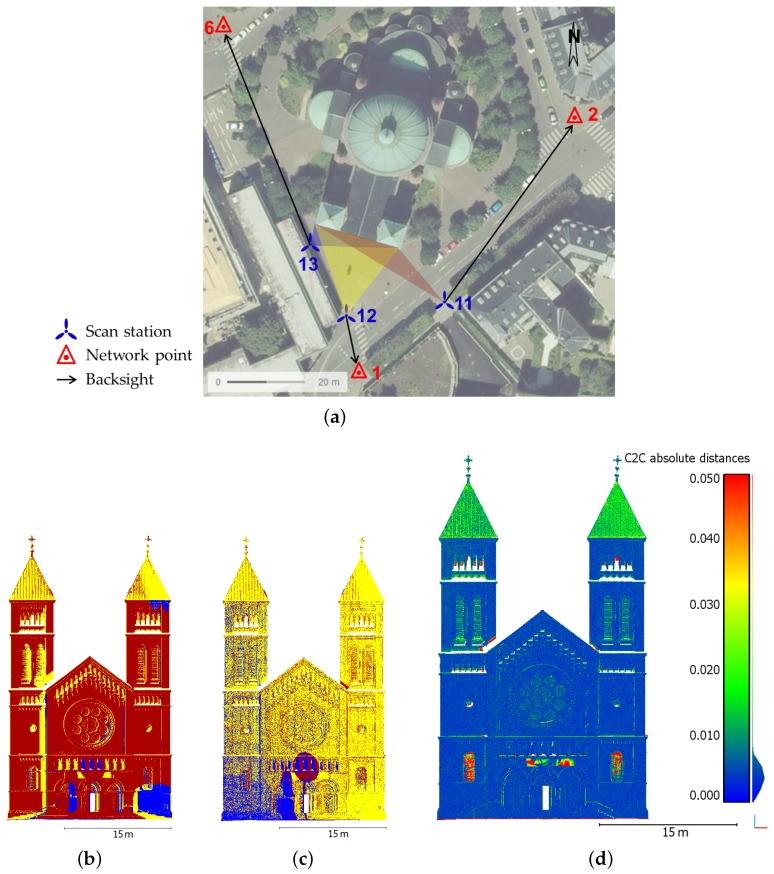
(**a**) Overview of the area around church facade; (**b**) Three point clouds acquired on the surveyed church facade without alignment refinement; (**c**) Point clouds finely registered after Iterative Closest Point (ICP); (**d**) Cloud-to-cloud comparison between FARO Focus^3D^ and Trimble SX10 point clouds of the facade. Deviations (unit: m) are projected on SX10 point cloud.

A first scanning project has been performed on the facade of a church which is located in Strasbourg, France. The facade has been captured using the Trimble SX10 from three different stations as shown in Figure 4a. The aim of this first project was to investigate the quality of the delivered point cloud on a detailed facade, and not to study the georeferencing capability. A network of known points available in the near environment of the church has still been used to roughly align the three point clouds in the field, as illustrated in Figure 4b which presents the three segmented point clouds. To correct the influence of potential uncertainties due to the survey and to really concentrate on the overall geometry, the point cloud alignment was refined by applying an algorithm based on the Iterative Closest Point (ICP) principle. This was made thanks to the manufacturer software Trimble RealWorks which delivered the lower remaining error after performing ICP in this project. Figure 4c depicts the result obtained after the three point clouds have been finely registered. With remaining alignment errors no larger than 1 cm between these individual point clouds, it clearly appears in this Figure that the data better overlap. The final segmented point cloud counts about 13 millions of points. Depending on the range between instrument and surveyed object, the *fine* resolution (stations 11 and 12) or the *standard* resolution (station 13) were used, leading to a global acquisition time of about 30 min.

In order to assess the quality of the global point cloud issued by the recent device, several comparisons are carried out with a reference dataset of the same church facade. The reference dataset has been acquired on the same day with a commonly used laser scanner Focus^3D^ from FARO. The reference point cloud counts about 50 millions of points captured from three scanning stations. Since the FARO device cannot be centered on a known point, these stations slightly differ from the ones used during the SX10 acquisitions. To perform comparisons between the Trimble SX10 and FARO Focus^3D^ point clouds, the free software CloudCompare is used. According to the computed cloud-to-cloud deviations presented in Figure 4d, a mean deviation smaller than 1 cm can be observed. However some high deviations appear in particular on the windows located in the lower part of the facade, but this can be explained by the noise caused by this reflective material. It should also be noticed that the high values visible above tympanum are caused by a banner which was present during the acquisitions. Finally, the presence of non-overlapping parts between both datasets due to slightly different viewing angles from the scan stations produce small deviations, which may be visible in particular on edges next to cornices, windows and pillars, and more generally on complex parts of a facade. Given that the deviations provided by a cloud-to-cloud comparison are non-signed, further comparisons have been performed in order to derive finer statistical values.

**Figure 5:**
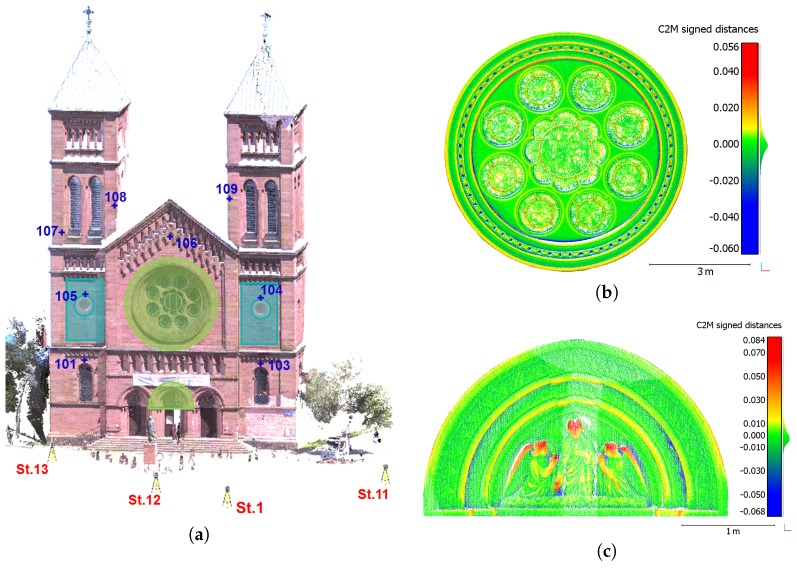
(**a**) Overview of the facade containing control points and locations of the areas selected for finer analyses; (**b**,**c**) Cloud-to-mesh comparisons performed on detailed areas between the SX10 point cloud and meshed parts of FARO Focus^3D^ point cloud: the rose window (**b**) and the tympanum (**c**). Deviations are in meters and projected on SX10 point cloud.

To gain a more reliable idea of the Trimble SX10 point cloud quality, a statistical analysis of the computed deviations has been performed based on some detailed and representative parts of the facade. The selected areas highlighted in Figure 5a are a rose window and a tympanum with moldings (green areas in the Figure), as well as planar surfaces (blue rectangular areas). Cloud-to-mesh distance computations are performed on these limited areas, which has the advantage of delivering signed values of the deviations unlike previous cloud-to-cloud comparisons. The Poisson surface reconstruction algorithm available in CloudCompare was applied on the dense point cloud acquired by FARO Focus^3D^ in order to create a reference mesh. This mesh and the areas segmented from the SX10 point cloud were finely registered using an ICP-like algorithm, delivering a final alignment error of about 9 mm and 1 cm respectively for the rose window and tympanum, and about 2 mm for planar surfaces.

Figure 5b,c show the deviations that were computed between the mesh and segmented point clouds for rose window and tympanum. These figures firstly illustrate the fine level of detail that is reached thanks to the SX10 point clouds. The resulting point clouds appear to be geometrically accurate since a standard deviation of about 2 mm is observed in both cases. As visible on both figures, the highest deviations are present in areas which are hardly accessible for the laser beam from the ground, producing grazing angles. It should also be noticed that the mesh creation process may have a small influence on the initial point cloud geometry, but this does not have much impact on the final analysis. Then regarding planar areas on the left and right parts of the facade (blue rectangular areas in Figure 5a), a standard deviation which varies between 3 and 4 mm with respect to adjusted planes is computed. These results are reliable considering the fact that the surveyed planar surfaces are made of stones which are thus not perfectly planar and aligned. The same statement is made while adjusting planes on the same segmented areas from the FARO Focus^3D^ point cloud, where standard deviations between 5 and 6 mm are observed. This difference in terms of deviation is mainly caused by the higher amount of points in the FARO Focus^3D^ segmented point clouds which are about twice as dense as those from the Trimble SX10. To complete these analyses, further comparisons performed on the same Trimble SX10 dataset but using other devices than the FARO laser scanner are presented in [38]. The computed deviations are very similar to those obtained here with the FARO Focus^3D^.

Finally, some control points were chosen on the facade as depicted in Figure 5a. Using a Leica TS02 total station, their 3D coordinates were determined in the local network partially illustrated in Figure 4a. These points were then measured thanks to the Trimble SX10 surveying facilities from station 1 in front of the facade (see Figure 5a). It should be noted that due to the height of the facade, it was impossible to physically mark these points on the edifice. The 3D deviations computed between the two surveys vary from 5 mm up to 2.5 cm. These results are acceptable given that the control points are natural features of the facade.

### 5.2. Assessment of a Directly Georeferenced Project

**Figure 6:**
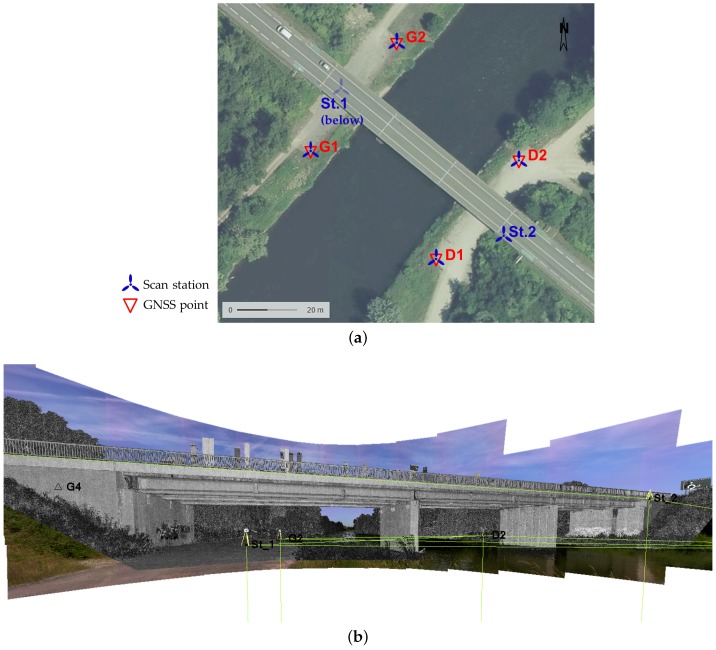
(**a**) Overview of the area around surveyed bridge with four of the Global Navigation Satellite Systems (GNSS) reference points; (**b**) Station view captured from Trimble Business Center office software. Georeferenced point cloud (in grey) and images are visible as seen from the considered station (station G1 in this case).

The second scanning project which is presented in this article involves a road bridge located near the Franco-German border in the Alsace region, France. A network of known points has been previously built in this area by determining the coordinates of some points with a GNSS receiver. Figure 6a presents an overview of the area containing reference points used during the project. This figure also shows that the four reference points below the bridge on the riverbank have been used as scanning stations. For each of these stations, the Trimble SX10 was set up on one considered reference point and oriented on the three others. To complete the point cloud of the bridge, two further scanning stations have been set up, under the bridge deck on one side and on the deck on the other. The new station coordinates have been determined through resection based each time on three visible references. Thus each of the six acquired point clouds is known in the coordinate system applied in this study, so that the whole scanning project is directly georeferenced. A station view from TBC software is presented in Figure 6b. On such a view, the user can visualize the georeferenced point clouds and pictures acquired from the selected station, as well as references and surveyed points.

The same scene was then acquired using a Leica ScanStation C10 which also makes it possible to directly georeference scanning projects. Compared to the Trimble SX10, targeting of individual points is not foreseen in the hardware surveying facilities. This is the reason why georeferencing is performed by stationing on a known point and scanning a small area around a specific target placed on another known point. Six scanning stations were also useful with the Leica C10 to capture not only the structure geometry but also some parts under and on the bridge deck. The *high resolution* parameter was chosen during Leica C10 acquisitions, corresponding to a point spacing of 5 cm at 100 m. In comparison, the Trimble SX10 point clouds have been acquired using the *standard* resolution parameter (5 mm @ 10 m) except for the station under the bridge where the *coarse* parameter (10 mm @ 10 m) has been used. After segmenting the datasets to keep only the considered structure and to remove the dense surrounding vegetation, Trimble SX10 whole point cloud counts about 9 millions of points.

**Figure 7:**
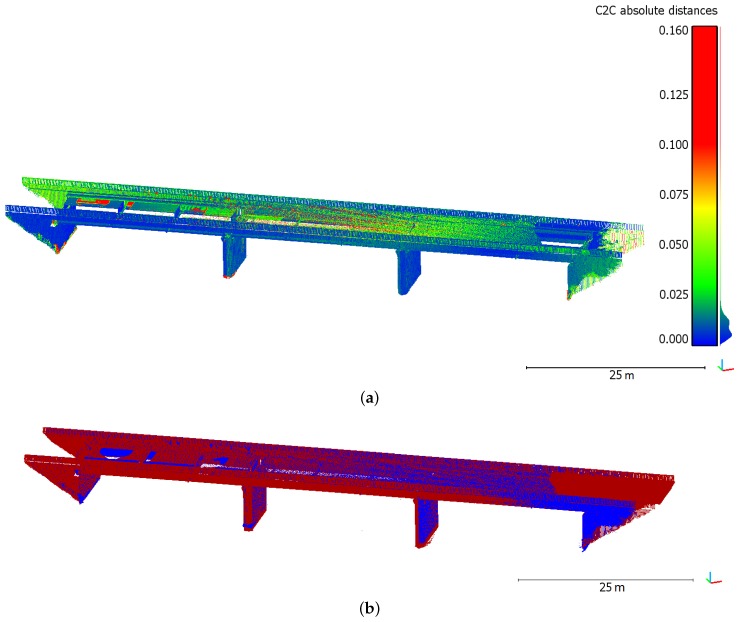
(**a**) Cloud-to-cloud comparison between Leica C10 and Trimble SX10 directly georeferenced point clouds of the bridge. Deviations (in m) are projected on SX10 point cloud; (**b**) Overview of both Leica C10 (in red) and Trimble SX10 (in blue) point clouds displayed together.

The aim of this second scanning project is mainly to assess the performed georeferencing. In the following comparisons, the segmented raw data are considered without refinement of the individual point clouds alignment. This is meant to avoid skewing the georeferencing if the initial registration is modified. Cloud-to-cloud comparisons using CloudCompare software have been carried out between Leica C10 and Trimble SX10 point clouds. Because the free stations for which the coordinates are computed by resection (see stations St.1 and St.2 in Figure 6a) were not located exactly on the same points during both scanning campaigns, hidden parts differ in both point clouds, in particular under the bridge deck. Such areas with little overlap between the two datasets may cause the computation of high cloud-to-cloud deviations which are not relevant. Besides, the amount and scattering of points acquired on the deck significantly vary between the two point clouds. The two main reasons which explain these differences are unavoidable and cannot be managed by the user: first the high road traffic on such a structure creates different masks between two acquisition campaigns; then the way grazing angles are managed and filtered by the instruments is very likely different for both devices. In order to better visualize the small deviations that are meaningful between the datasets, points belonging to previously described areas and presenting obviously too high deviations have been filtered. The result of cloud-to-cloud distance computation on the remaining bridge structure is shown in Figure 7a.

A first look at the comparison shows that no significant geometrical deviations of the structure are observed between both datasets. A mean deviation smaller than 2 cm can be derived from this analysis for the bridge main structure, where points belonging to upper and lower parts of the deck have been filtered. A closer look at the comparison result provides relevant information about the georeferencing. As a matter of fact, deviations of the same order of magnitude are observed on the opposite sides of each bridge abutment. Since this appears on opposite sides with respect to the road axis, it illustrates that C10 and SX10 point clouds have a slightly different orientation due to their respective georeferencing. While superimposing both raw segmented point clouds, this trend becomes clearer. Figure 7 shows that on the presented side, the blue dataset (Trimble SX10) stands out on the right abutment whereas the red one stands out on the left part. This is also visible from the other side of the bridge point cloud. On these particular endpoints of the bridge, computed deviations do not exceed 6 cm and are almost the same for each opposite abutment. However the overall structure of the point clouds do not suffer from any significant distortion. Regarding the bridge length, the orientation deviation between both bridge axes is acceptable. The presence of this deviation highlights how important the reference targeting performed to compute georeferencing is. The result obtained underlines that georeferencing mainly depends on the network quality and can vary while using different instruments.

**Figure 8:**
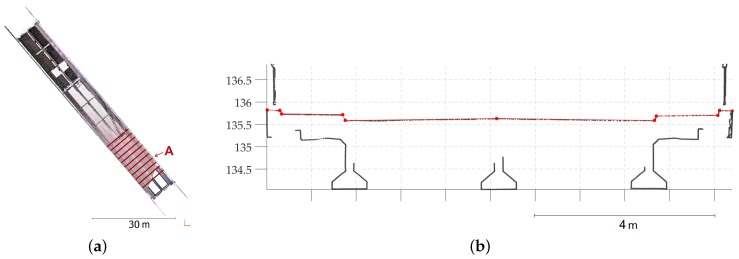
(**a**) Top view of the bridge point cloud with locations of the investigated sections (red lines); (**b**) Section A performed in Trimble SX10 point cloud and displayed together with the tacheometric profile data (in red).

To complete the study in particular on the bridge deck, the Trimble SX10 point cloud has been confronted to a previous total station survey. Altitudes of some significant points of the deck have been compared to existing georeferenced profiles. The considered points which have been manually selected in the point cloud are the road axis, the limits of the pavement and the sidewalks. Based on comparisons performed on 11 successive profiles 2 m apart (see Figure 8a), the mean altitude deviation computed was about 7 mm. A detailed analysis of each profile shows that the highest deviations are mainly observed on the last sections near the middle of the bridge deck because of the lower point density. Points acquired on this part of the deck are more scattered due to grazing angles and thus more noise is present in the point cloud. For the remaining sections, the deviations are randomly distributed between negative and positive values and no systematic error is observed. Since GNSS has been used to observe the initial network (Figure 6a), the achieved georeferencing could not offer better results. To complete the analysis, Figure 8b presents a section carried out in the point cloud displayed together with its corresponding tacheometric profile, in red. It can be seen that the point cloud geometry accurately follows the previous surveying measurements.

### 5.3. Uncertainty Analysis Related to Object Color and Materials

It has been shown many times in the literature that the properties of the scanned object such as color, roughness or reflectivity [39,40] and even the moisture [41] of the scanned material can have an influence on the distance measured by laser scanners. The incidence angle of the laser beam on the reflected surface is also of relevance [8]; nevertheless this specific point was not examined during our tests.

**Figure 9:**
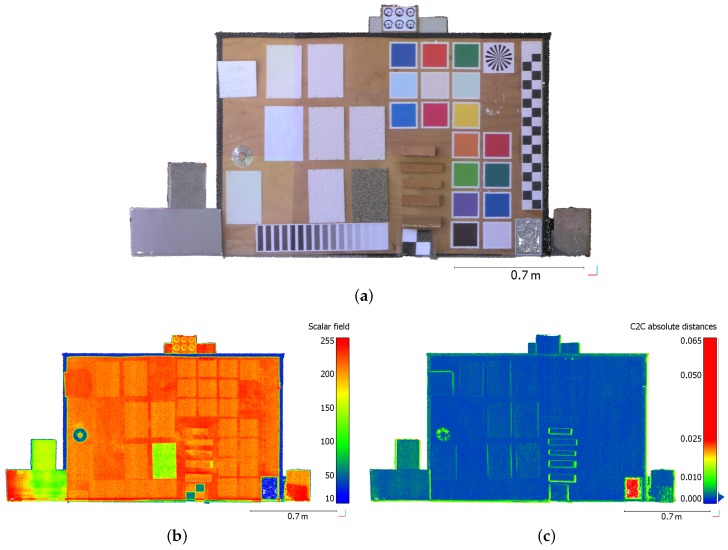
(**a**) Trimble SX10 colorized point cloud of a board composed of various color and material samples; (**b**) Visualization of intensity values returned by the laser scanner; (**c**) Cloud-to-cloud comparison between FARO Focus^3D^ and Trimble SX10 point clouds of the same board. Deviations (unit: m) are projected on SX10 point cloud.

Since the previous scanning projects were carried out in dry weather, the performed laboratory analyses mainly focus on the type of material, as well as on its color and reflectivity. A board composed of various samples has been scanned with the Trimble SX10 at a distance of about 9 m. Using the *superfine* scanning resolution, the point cloud representing the sample board counts slightly more than 1 million points. On this colorized point cloud shown in Figure 9a, it appears that the board is composed of color samples on the right part, and different kinds of wood samples in the middle. On the left part, samples with different textures and roughnesses can be seen. Flat black and white targets have also been placed in the scene, as well as a mirror in the lower right corner or bricks and cement blocks on each side of the board. Regarding the intensity values provided by the laser scanner and depicted in Figure 9b, it is clearly visible that black and reflective materials present low intensity values. As a matter of fact, reflective materials such as the mirror surface or the compact disk cause a specular reflection of the laser beam whose few signal is returned back to the TLS. Then, since black materials are very absorbing, the amount of signal they reflect back to the scanner is also small. Considering the remaining samples, it appears that the color variations do not really influence the amount of returned intensity.

To better investigate the geometry of the resulting point cloud by comparing it to data coming from another laser scanner, the board was also scanned with a FARO Focus^3D^ placed at the same location. Both point clouds were first aligned using an ICP-like algorithm (alignment error: 4 mm), and were then compared using the CloudCompare software. Figure 9c shows a cloud-to-cloud comparison between both Trimble and FARO point clouds, whereby the computed distances are projected on the Trimble SX10 point cloud.

It can be seen on this figure that the geometries of the point clouds are very similar since most of the deviations are smaller than 5 mm, which is within the order of magnitude of the alignment error. The main difference between both datasets is visible on the mirror, since no points were measured by the FARO Focus^3D^ on such a highly reflective surface. Deviations can also be observed on the edges of the board which are covered with large black tape. This means that the filtering of highly reflective (mirror) and very absorbing (black patterns) surfaces is probably managed differently in both devices. Deviations observed on the edges of wood samples for example, are mainly due to the alignment between both datasets and to the grazing angles of the laser beam on these parts.

Regarding color variations, this has obviously no significant influence on the range measurement. A small amount of measurement noise is only visible on black patterns, and this amount is not exactly the same between both datasets. Besides, intensity values provided by each laser scanner are very similar for the data acquired on the considered samples. One can assume that filters are applied on the observations to reduce the influence of object color on the measured data.

All the previously assessed projects have shown a high similarity between Trimble SX10 data and datasets issued from other laser scanners. The results of these comparisons have proved that the device is efficient and as reliable as other commonly used instruments, and this validates the announced performance. Some of the major characteristics of the Trimble SX10 scanning total station are summarized and discussed in the next section.

## 6. Discussion

It is obvious that total stations are nowadays becoming smarter thanks to the integration of new capabilities and sensors into the hardware. This trend corresponds to a new way of thinking and performing surveying, aided by constant technological evolutions. Meanwhile laser scanning is a more and more appreciated solution for the fast acquisition of three dimensional geometry. In this context, the concept of scanning total station has been recently introduced to qualify instruments devised not only for scanning purposes, but also to perform conventional tacheometric tasks. The Trimble SX10 scanning total station belongs to this category and is intended for use in traditional surveying as well as in larger laser scanning projects. The absence of an optical telescope replaced by a powerful camera technology contributes to its originality. This daring decision to remove the telescope is made possible thanks to the use of a remote controller as well as of a set of cameras, and it clearly benefits the measuring rate of the device. More generally, the versatility of this new generation of instruments probably represents its most valuable aspect since a wide variety of measurement tasks can be carried out with only one unique instrument. This has already been suggested in the past years such as in the conclusion of [7], where the authors were regretting the absence of facilities other than resection for the device location during georeferencing with scanning devices.

Beyond competitiveness and verified high geometrical accuracy, the Trimble SX10 singular all-in-one design and its on-board camera technology among other features are significant added values. However depending on the surveying project, a common total station may sometimes be sufficient. Even if direct georeferencing is rather easy to perform with such an instrument, it may not be the most adapted solution in some particular scanning projects such as complex buildings for example. This is also true for common laser scanning projects, to which a total station survey may be preferred depending on the required deliverable. The high number of provided facilities should obviously not bypass a careful preparation and analysis of the necessary data by the user before acquisition. The absence of a telescope which may be confusing for uninitiated users also corresponds to a digital evolution that will probably impact most of the future device developments. Regarding all the previously mentioned attributes, the Trimble SX10 definitely belongs to the next generation of equipment for land surveyors and people related to field surveying in their specific research topics.

## 7. Conclusions

The aim of this article was to give an overview of the major capabilities of the recent Trimble SX10 scanning total station. Based on a review of existing instruments providing similar functionalities, the innovative device was first put in the current context of surveying instrument development. Some major technical properties of the Trimble SX10 were confronted to those of currently commercialized IATS as well as TLS. Based on this purely technical comparison, the device is competitive considering the multiple facilities it delivers in one unique piece of hardware. Advantages such as the possibility to document a project with pictures or to capture georeferenced point clouds and panoramas have been highlighted, as well as its originality which is based on the absence of a telescope. The quality of the produced data was then investigated through a set of acquisitions carried out under various conditions. Most of the Trimble SX10 capacities were tested, not only scanning performances but also surveying facilities. Their use has been reported through different studies presented in this article, such as building facade or bridge survey. It is important to note that the presented case studies are real projects for which the use of such an “all-in-one” instrument could bring an added value. To assess the quality of the produced data, they were confronted to results issued by similar instruments and methods. Scanning projects in particular were compared to datasets produced with other TLS. Direct georeferencing also belongs to the investigated capabilities through a civil engineering structural survey. A finer assessment of the real precision achievable using the Trimble SX10 would require the performance of experiments on a specifically designed calibration benchmark. Nevertheless, the few case studies presented throughout the article correspond to typical applications which could benefit from the facilities offered by this particular instrument. Based on these experiments, the results obtained are valid compared to those delivered by the instruments used in our surveys. For these reasons one can conclude that the Trimble SX10 scanning total station meets the needs of users in terms of convenience and precision for common surveying tasks, depending on the expectations required by the project. Due to the advantages related to a powerful and competitive multi-sensor system, it also answers to further needs and enlarges the choice of users in the range of smart surveying instruments.

## Figures and Tables

**Table 1:** Overview of scanning total stations with some of their technical specifications according to manufacturers.

	Topcon IS-3	Leica MS60	Trimble SX10
	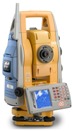	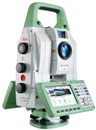	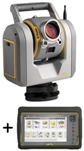
Date of release	2011	2015	2016
Angular accuracy	1”, 3” or 5”	1”	1”
EDM maximum range	5000 m (prism)	10,000 m (prism)	5500 m (prism)
	2000 m (non prism)	2000 m (non prism)	800 m (non prism)
EDM accuracy (prism)	2 mm + 2 ppm	1 mm + 1.5 ppm	1 mm + 1.5 ppm
EDM accuracy (non-prism)	10 mm + 10 ppm	2 mm + 2 ppm	2 mm + 1.5 ppm
**Imaging**			
Number and kind of cameras	wide-angle + coaxial	overview + telescope	overview + primary + coaxial
Resolution	1.3 megapixel	5 megapixel	5 megapixel
Frame rate	up to 10 Hz	up to 20 Hz	up to 15 Hz
**Scanning**			
Maximum rate	20 pts/s	1000 pts/s @ 300 m	26,600 pts/s
Maximum range	2000 m	1000 m (limited to 1 Hz)	600 m
Scanning range noise	-	1 mm @ 50 m	1.5 mm @ 50 m

**Table 2:** Some specifications of Trimble SX10 on-board cameras according to manufacturer.

Specifications	Overview Camera	Primary Camera	Telescope Camera
Position relative to EDM axis	parallel	parallel	coaxial
Field of view	54°	12°	2°
Pixel size @ 50 m	20 mm	4.4 mm	0.88 mm

**Table 3:** Point spacings achievable with the Trimble SX10 depending on scanning density.

	Coarse	Standard	Fine	Superfine
Point spacing @ 10 m	10 mm	5 mm	2 mm	1 mm

**Table 4:** Comparison of some technical properties of the three scanning devices used in the article, according to manufacturer specifications.

	Leica ScanStation C10	FARO Focus^3D^ X330	Trimble SX10 Scanning Total Station
	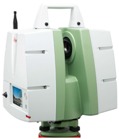	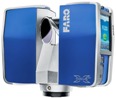	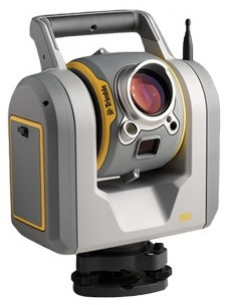
Measurement principle	time-of-flight	phase-shift	time-of-flight
Scanning rate	up to 50 kHz	up to 976 kHz	up to 26.6 kHz
Max. measurement range	300 m	330 m	600 m
Accuracy criterion *	*Accuracy of single dist. measurement:* 4 mm @ 1–50 m (one sigma)	*Ranging error:* 2 mm @ 25 m (one sigma)	*Distance measurement accuracy (DR mode):* 2 mm + 1.5 ppm
Range noise *	2 mm	max. 0.5 mm @ 25 m	1.5 mm @ 50 m
Point spacing	<1 mm minimum through full range *	from 1.5 mm to 49 mm @ 10 m	from 1 mm to 10 mm @ 10 m
Laser wavelength	532 nm	1550 nm	1550 nm
Date of release	2009	2013	2016

* terminology and values directly derived from technical datasheets of respective manufacturers.

**Table 5:** Percentage of points contained in each 100 m portion around the scanner station.

Range Interval	Number of Points	Percentage According to Whole Point Cloud
<100 m	412,861	32.95%
100 m–200 m	446,500	35.64%
200 m–300 m	235,924	18.83%
300 m–400 m	71,201	5.68%
400 m–500 m	38,714	3.09%
500 m–600 m	33,311	2.66%
600 m–700 m	11,558	0.92%
700 m–800 m	1313	0.10%
800 m–900 m	1425	0.11%
>900 m	125	0.01%

## Abbreviations

The following abbreviations are used in this manuscript:

TLS	Terrestrial Laser Scanner
GNSS	Global Navigation Satellite System
EDM	Electronic Distance Measurement
IATS	Image-Assisted Total Station
COGO	Coordinate Geometry
TBC	Trimble Business Center
ICP	Iterative Closest Point
